# Multi-trait association analysis reveals shared genetic architecture between lung cancer and cardiometabolic diseases

**DOI:** 10.1016/j.isci.2025.114129

**Published:** 2025-11-19

**Authors:** Qiong Lyu, Xuan-Yu Wang, Erhong Chen, Chanjuan Sun, Zhengang Qiu, Yingyu Xie, Ping He

**Affiliations:** 1 Department of Pathology, The First Affiliated Hospital of Guangzhou Medical University, Guangzhou, China; 2 College of Traditional Chinese Medicine, Tianjin University of Traditional Chinese Medicine, Tianjin 301617, China; 3 Tianjin Key Laboratory of Modern Chinese Medicine Theory of Innovation and Application, Tianjin University of Traditional Chinese Medicine, Tianjin 301617, China; 4 Department of Oncology, The Sihui People’s Hospital, Zhaoqing, China; 5 Department of Oncology, Zhujiang Hospital, Southern Medical University, Guangzhou, China; 6 Department of Oncology, The First Affiliated Hospital of Gannan Medical University, Ganzhou, Jiangxi, China

**Keywords:** Health sciences, Medicine, Medical specialty, Internal medicine, Cardiovascular medicine, Oncology, Bioinformatics, Medical informatics

## Abstract

Lung cancer (LC) frequently coexists with cardiometabolic diseases (CMDs), complicating clinical management, but their shared genetic architecture remains largely unknown. Here, we analyzed genome-wide association study (GWAS) statistics for LC and 36 cardiometabolic traits and diseases (CMTs) to determine genetic correlations and shared biological pathways. We further explored underlying mechanisms through the analysis of bulk and single-cell RNA sequencing data and identified potential therapeutic candidates using drug-gene interaction databases. Significant genetic associations were revealed between LC and 16 CMTs, including subarachnoid hemorrhage, peripheral arterial disease, heart failure, and physiological traits (Padj <0.05). The shared genes were identified as enriched in lipid and cholesterol metabolism. Notably, monocyte-derived macrophages (mo-Macs) in lung adenocarcinoma exhibited M2-like polarization under high cholesterol metabolism and rosuvastatin and lovastatin were identified as potential drugs for LC-CMD comorbidities. Our findings demonstrate a role for cholesterol metabolism in LC-CMD comorbidities, offering insights into the underlying mechanisms and potential therapeutic strategies.

## Highlights

• Lung cancer (LC) shares genetic correlation with cardiometabolic traits and diseases (CMTs)
• Shared genetic variants and genes involve cholesterol and lipid metabolism and immune pathways
• Macrophages mediate comorbidities through cholesterol-driven M2-like polarization
• Statins show therapeutic potential for comorbidities by targeting cholesterol metabolism

## Graphical abstract

**Figure:**
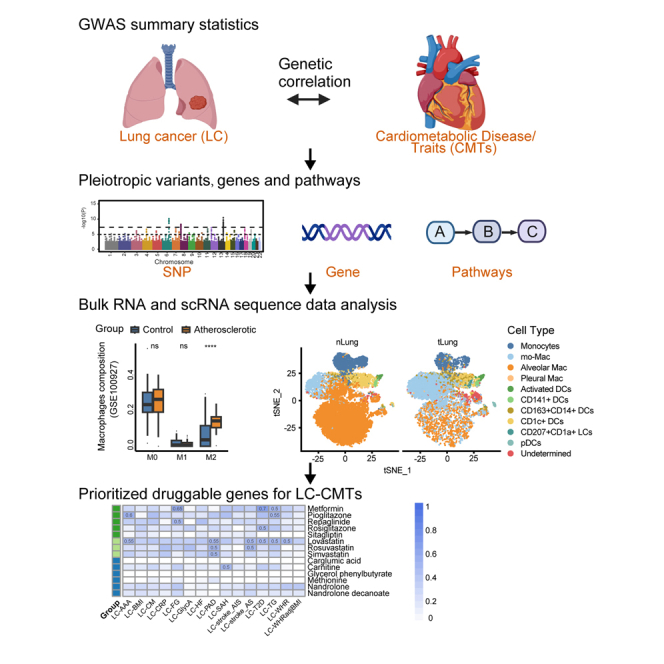

## Introduction

With almost 2.5 million new cases and 1.8 million deaths from lung cancer (LC) in 2022, it was the most prevalent cancer diagnosed and the leading cause of cancer-related deaths.^1^ Despite the fact that advancements in early cancer detection and effective anticancer therapies have improved the survival rates of patients with LC, the coexistence of cardiometabolic diseases (CMDs) has become a significant concern.^2^^,^^3^ Several factors contribute to this comorbidity, including organ and tissue toxicity from cancer therapies,^4^^,^^5^^,^^6^^,^^7^ as well as common risk factors like smoking and obesity, while the shared genetic determinants between these two conditions remain largely unknown.

Cardio-oncology has garnered increased concern in recent years, particularly regarding the comorbidity of LC and CMDs.^8^^,^^9^^,^^10^^,^^11^^,^^12^ Epidemiological studies have shown that survivors of LC have a significantly higher risk of atrial fibrillation, ischemic heart disease, heart failure (HF), and stroke compared to controls.^8^^,^^13^^,^^14^ Additionally, the association between metabolic disorders and an increased risk of LC has also been reported, including pre-diabetes, type 2 diabetes (T2D), and metabolic dysfunction-associated steatotic liver disease.^2^^,^^15^ Conversely, some preclinical studies have suggested that certain oncometabolites, such as D-2-hydroxyglutarate, may promote HF.^16^ However, little research has focused on the genetic associations of LC and CMDs and the integrated shared genetic architecture remains largely unexplored.

Macrophages are the primary immune cells in arteriosclerosis (AS), which serves as the underlying basis for various CMDs.^17^ Additionally, they are important cellular components of the tumor microenvironment in LC.^18^ Metabolic reprogramming can influence the polarization of macrophages, thereby affecting their function: M1 macrophages promote inflammation, while M2 macrophages inhibit inflammation and are associated with stabilizing atherosclerotic plaques.^19^ In this study, we further explore the role of macrophages in contributing to comorbidities of LC and CMDs by combining bulk and single-cell RNA (scRNA) sequencing (scRNA-seq) data from both conditions.

**Figure 1:**
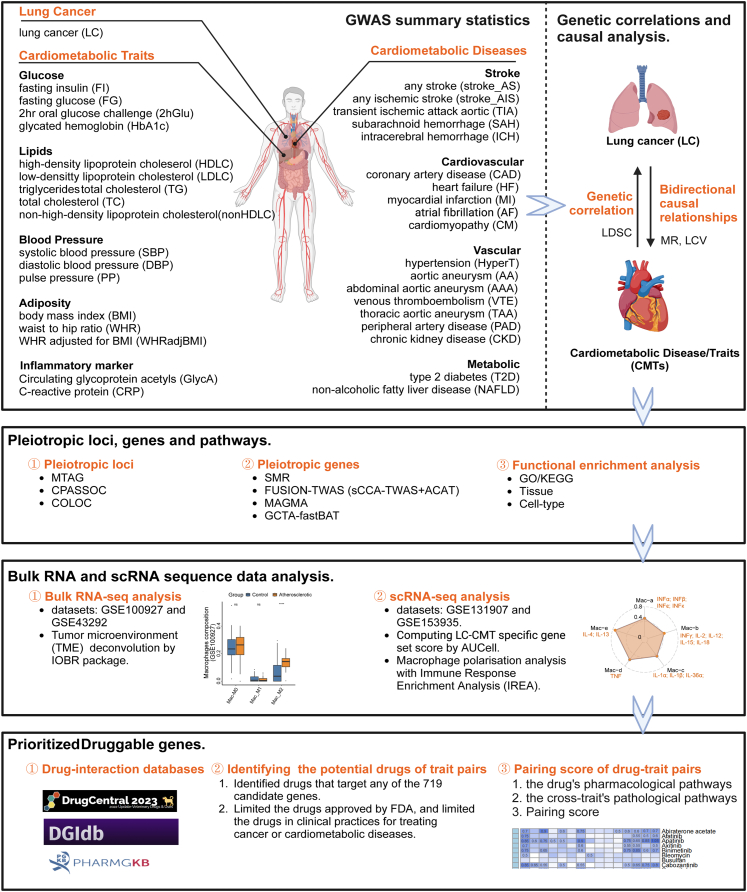
The overall flow chart of this study GWAS statistics for LC and 36 CMTs was collected to assess genetic correlations and bidirectional causality, followed by cross-trait meta-analyses (MTAG, CPASSOC) and colocalization to identify shared pleiotropic variants. Then, gene-based analyses and gene set enrichment were employed to reveal shared genes and pathways, while microenvironment deconvolution and immune response enrichment analysis were conducted to characterize the shared biological mechanisms. Finally, drug-gene interaction databases were leveraged to identify candidate drugs for both conditions.

Genome-wide association studies (GWASs) conducted with extensive sample populations have identified variants associated with LC and cardiometabolic traits and diseases (CMTs), making it possible to investigate the shared genetic mechanisms underlying these comorbidities. In this study, we incorporated GWAS summary statistics, as well as bulk and scRNA-seq data, to further investigate the shared genetic architecture and biological mechanisms. First, we comprehensively collected the GWAS statistics for LC and 36 CMTs and examined the genetic correlation and bidirectional causal relationship between LC-CMTs. Next, we employed two cross-trait meta-analyses (MTAG and CPASSOC) and colocalization analysis to identify the shared pleiotropic variants. We then conducted four gene-based analyses and gene set enrichment analysis to find the shared genes and biological pathways. To further characterize biological pathways involved in both diseases, we performed microenvironment deconvolution analysis for the bulk RNA sequencing (RNA-seq) data from atherosclerotic plaque, as well as the immune response enrichment analysis (IREA) on the scRNA-seq data of LC. Finally, we utilized the three largest drug-gene interaction databases to identify candidate drugs for both conditions. A schematic overview of the study is presented in Figure 1.

## Results

### Genetic correlation and causal inference

**Figure 2:**
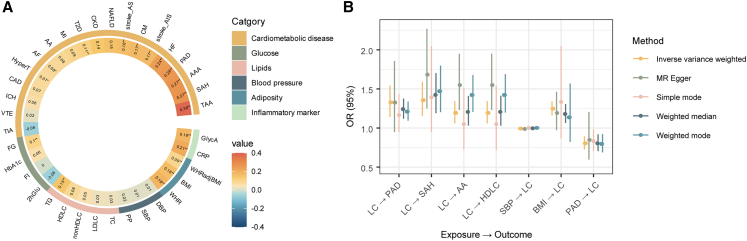
The comprehensive landscape of genetic correlation across LC and CMTs (A) The heatmap showing genetic correlation calculated by LDSC. The color and the number inside the squares indicate the correlation. The statistical significance is denoted by asterisks: ∗*p* < 0.05; ∗∗FDR-adjusted *p* (Padj) < 0.05. (B) Bidirectional causal relationship between lung cancer and CMTs determined through MR analysis. The results meeting *p* < 0.05 after FDR correction are displayed, with detailed information available in Table S3. stroke_AS, any stroke; stroke_AIS, any ischemic stroke; CAD, coronary artery disease; HyperT, hypertension; MI, myocardial infarction; CKD, chronic kidney disease; AF, atrial fibrillation; CM, cardiomyopathy; TIA, transient ischemic attack; VTE, venous thromboembolism; ICH, intracerebral hemorrhage; NAFLD, non-alcoholic fatty liver disease; FI, fasting insulin; FG, fasting glucose; 2hGlu, 2h oral glucose challenge; HbA1c, glycated hemoglobin; LDLC, low-density lipoprotein cholesterol; TC, total cholesterol; nonHDLC, non-high-density lipoprotein cholesterol; SBP, systolic blood pressure; DBP, diastolic blood pressure; PP, pulse pressure; WHRadjBMI, WHR adjusted for BMI; GlycA, circulating glycoprotein acetyls.

Positive correlations between LC and CMTs are indicated by the genome-wide association calculated by linkage disequilibrium score regression (LDSC) (Figure 2A). Among the correlations that remain significant after strict Benjamini-Hochberg adjusted (Padj <0.05), the most pronounced are observed with thoracic aortic aneurysm ([TAA] r_g_ = 0.38, *p* = 0.009, Padj = 0.026), followed by several other conditions such as subarachnoid hemorrhage ([SAH] r_g_ = 0.27, *p* = 0.002, Padj = 0.006), abdominal aortic aneurysm ([AAA] r_g_ = 0.27, *p* = 9.24 × 10^−8^, Padj = 8.31 × 10^−7^), peripheral artery disease ([PAD] r_g_ = 0.26, *p* = 4.73 × 10^−5^, Padj = 2.84 × 10^−4^), and HF (r_g_ = 0.24, *p* = 6.17 × 10^−5^, Padj = 3.18 × 10^−4^). For the correlations with physiological traits, C-reactive protein (CRP) shows a positive correlation with LC (r_g_ = 0.21, *p* = 7.53 × 10^−11^, Padj = 2.72 × 10^−9^). Adiposity traits also display positive correlations with LC, with waist-to-hip ratio (WHR) (r_g_ = 0.18, *p* = 7.52 × 10^−10^, Padj = 1.35 × 10^−8^) and body mass index (BMI) (r_g_ = 0.16, *p* = 5.86 × 10^−9^, Padj = 7.03 × 10^−8^) being notable examples (Table S2). Replication analysis using the GWAS statistic from FinnGen R12 and LC subtypes revealed similar patterns, suggesting comorbidity between LC and CMTs (Figure S1).

Despite genetic correlations resulting from complex pleiotropic effects, Mendelian randomization (MR) analysis employing various models overcomes confounders and provides causal inference (Figure 2B, Table S3). In our study, alongside the established risk factor BMI (inverse-variance weighted method [IVW, odds ratio [OR] [95% confidence interval (CI) = 1.25 [1.16–1.34], *p* = 2.72 × 10^−9^), four MR analysis models indicated a bidirectional causality between LC and PAD. Specifically, LC that was genetically predicted was linked to a higher risk of PAD (IVW, OR [95% CI] = 1.33 [1.14–1.54], *p* = 2 × 10^−4^). Conversely, PAD was causally protective against LC (IVW, OR [95% CI] = 0.80 [0.72–0.89], *p* = 4.72 × 10^−5^). Additionally, LC was implicated as causally related to SAH (IVW, OR [95% CI] = 1.36 [1.16–1.59], *p* = 1.9 × 10^−4^). The results from the latent causal variable (LCV) analysis suggested a partial causal effect of LC on PAD (genetic causal proportion = 0.66, *p* = 1.96 × 10^−5^) (Table S4). Replication MR analyses using the additional GWAS statistics again showed that BMI was associated with an increased risk of LC (IVW, OR [95% CI] = 1.29 [1.19–1.40], *p* = 2.01 × 10^−9^), with significant associations in lung squamous cell carcinoma (LUSC) (IVW, OR [95% CI] = 1.50 [1.35–1.68], *p* = 1.57 × 10^−13^) and small cell lung cancer (SCLC) (IVW, OR [95% CI] = 1.72 [1.47–2.02], *p* = 3.28 × 10^−11^), but not in LUAD (Figure S1; Tables S5 and S6). The positive genetic association between LC and low-density lipoprotein cholesterol (HDLC), aortic aneurysm (AA), PAD, and SAH was also confirmed by the replication analysis, with varying significance levels among different LC subtypes.

### Cross-trait loci and causal variants

**Figure 3:**
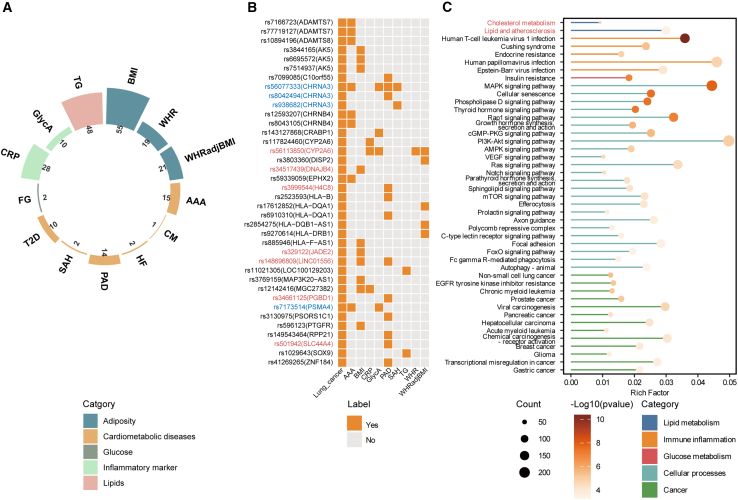
The comprehensive landscape of pleiotropic associations across LC and CMTs (A) The number of independent SNPs shared between LC-CMTs trait pairs; detailed information available in Table S7. (B) The heatmap displays the colocalization results between LC-CMTs trait pairs. Red font: SNPs known to be associated with LC; blue font: SNPs known to be associated with lung function. (C) KEGG enrichment analysis of the collection of shared genes, arranged according to biological processes. Signaling pathways associated with lipid and cholesterol metabolism are highlighted in red.

Before conducting cross-trait meta-analysis, we examined the distribution of sample size for each trait, a crucial factor in determining the power of GWASs. LC had a sample size of 85,716, while other diseases or traits ranged from 19,646 to 1,996,991. In the datasets of paired LC and CMTs, valid common SNPs numbered approximately 5.5 million, except for LC-SAH (approximately 3.8 million), ensuring the robustness of the results (Figure S2). For CPASSOC analysis, 10 trait pairs showed comparable numbers of significant SNPs with >80% overlap. However, six pairs (LC-AA, LC-CAD, LC-ICH, LC-stroke_AIS, LC-TIA, and LC-HDLC) demonstrated substantial heterogeneity, with SHet identifying >80% of significant SNPs, while the overlap between methods was <10% (Figure S3). This aligns with previous findings that SHom is more powerful when heterogeneity is absent, while SHet accommodates trait heterogeneity effects. By conducting meta-analysis using both MTAG and SHet methods from CPASSOC, we discovered 227 shared variants across 13 of 16 LC-CMTs trait pairs (Table S7, kb = 500, and r2 = 0.2). The remaining 3 pairs showed no significant associations: the LC-TAA trait pair failed quality control of MTAG, while no significant SNPs were detected in the LC-stroke_AIS and LC-stroke_AS trait pairs. In general, LC exhibits the highest number of shared independent SNPs with BMI (*n* = 55), followed by triglycerides (TGs) (*n* = 48) and CRP (*n* = 28) (Figure 3A). With more stringent clumping thresholds, the number of significant independent SNPs was reduced to 145 for the 13 LC-CMTs trait pairs (kb = 1,000, and r2 = 0.001).

Next, we retrieved the variations within 500 kb of the index SNP to determine the shared causal SNPs by colocalization method (Figure 3B). Among the 38 causal variants that were shared by at least one pair of traits, seven SNPs were known variants for LC: rs56113850(CYP2A6), rs34517439(DNAJB4), rs3999544(H4C8), rs329122(JADE2), rs148696809(LINC0156), rs34661125(PGBD1), and rs501942(SLC44A4). Cytochrome P450 2A6 (CYP2A6) was annotated from 2 SNVs in 4 trait pairs, in which rs56113850 was causal to 3 trait pairs, and metabolizes up to 70% of nicotine into cotinine via C-oxidation, and genetic variants affecting CYP2A6 activity were related to LC and AS.^20^^,^^21^ In addition, we found four SNPs known for lung function: rs56077333, rs8042494, rs938682 (CHRNA3), and rs7173514(PSMA4). Genetic studies have previously reported SNPs at chromosome 15q25.1, which includes the nicotinic receptor subunit genes CHRNA5-CHRNA3-CHRNB4 linked to the risk of LC and PAD,^22^ of which rs56077333 located in CHRNA3 was found to be shared in five traits in our study. Cross-trait meta-analysis with larger sample sizes offers enhanced statistical power to uncover more SNPs associated with both LC and CMTs. For example, our analysis revealed six SNPs (rs2523593, rs17612852, rs6910310, rs2854275, rs9270614, and rs885946) within HLA genes, which encode the major histocompatibility complex and play a critical role in antigen presentation and recognition, as being linked to susceptibility to both LC and PAD.

### Shared gene enrichment analysis indicated that lipid metabolism was enriched in LC

In addition to annotating SNPs by proximity using ANNOVAR, we applied four additional transcriptome-wide analysis methods to identify disease-associated genes: TWAS-Fusion, SMR, MAGMA, and GCTA-fastBAT. Genes significantly identified by all four methods were categorized as disease-related genes, meeting the criteria of *p* < 0.01 after false discovery rate (FDR) correction, resulting in a total of 731 genes. Among these genes, 92 were associated with at least 3 LC traits (Table S8).

Combining genes identified through any of the four methods to gain a comprehensive view of biological pathways allows us to analyze genes associated with specific trait pairs to obtain detailed insights for each condition (Figure 3C). As anticipated, we observed enrichment of tumor-related pathways such as non-small cell LC, gastric cancer, and prostate cancer. Classic signaling pathways like AMPK, VEGFR, and Notch, previously linked to LC development, were enriched. Furthermore, we noted enrichment in immunity, cholesterol metabolism, lipids, and atherosclerosis. Subsequently, we conducted gene set enrichment analysis based on disease pairs, focusing on the top 10 enriched signaling pathways in each LC-CMTs pair. Immune-related pathways were notably enriched in various LC-CMTs trait pairs, while CMT-related traits like cholesterol metabolism were enriched in LC-TG and LC-AAA (Figure S4). In the replication analysis, cholesterol metabolism pathways were also enriched in LC-CMTs trait pairs (Figure S5). Extended analysis of LC subtypes revealed that cholesterol metabolism and lipid and atherosclerosis signaling pathways were enriched in LUAD, LUSC, and SCLC (Figure S6), suggesting that lipid and cholesterol metabolism may play a significant role in these LC subtypes as well. Notably, the cholesterol metabolism pathway showed the strongest significance in LUAD (LUAD, *p* = 2.52 × 10^−7^; LUSC, *p* = 7.60 × 10^−6^; SCLC, *p* = 7.27 × 10^−5^). We created a circular dendrogram for LC-CMTs using the link between the summarized SNPs and genes, which illustrates the genes and variations that are shared by trait pairs (Figure S7).

### Tissue and cell enrichment analysis suggested that macrophages are involved in LC-CMTs

**Figure 4:**
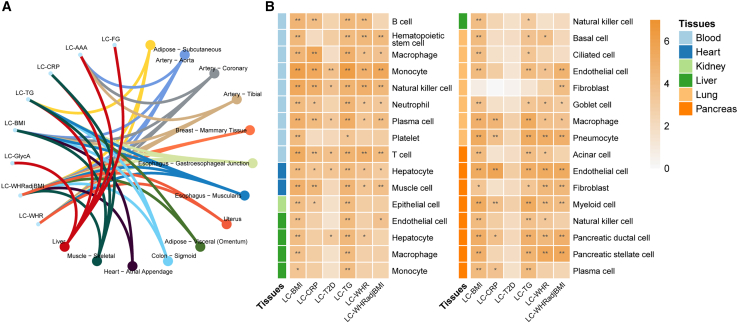
Tissue and cell-type specificity inferred from the shared signals between LC and CMTs (A) Enriched tissue types of each trait pair by TSEA. (B) Enriched tissue-specific cell types of each trait pair by CSEA.

The shared genes may function in certain tissues and cell types more specifically. We performed tissue-specific enrichment analysis (TSEA) and cell-type-specific enrichment analysis (CSEA) for the LC-CMTs shared genes reported by any of the four methods. For the TSEA, we discovered that the artery, adipose tissue, and liver were significantly enriched in at least three LC trait pairs, while the heart atrial appendage was significantly enriched in adiposity-related LC trait pairs (Figure 4A). In the CSEA, macrophages were the most enriched cell, including macrophages from blood, liver, and lung (Figure 4B), indicating that they serve as the center for LC and cardiometabolic processes. LC-TG and LC-BMI share the most enriched tissue-specific cells and have great similarities. In summary, these results, together with the gene and pathway findings, highlight the important roles of lipid metabolism and immunity in LC and CMTs.

### Enhanced cholesterol metabolism of mo-Macs in LUAD

**Figure 5:**
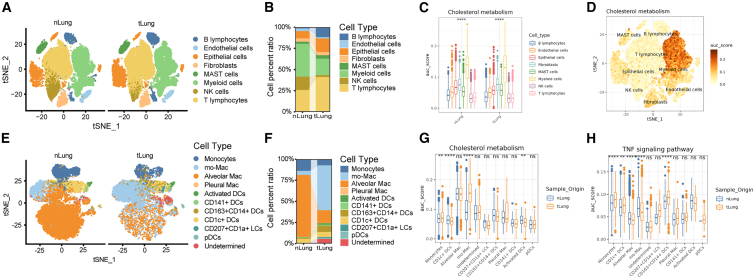
Enhanced cholesterol metabolism of mo-Mac in LUAD (A) The t-distributed stochastic neighbor embedding (t-SNE) plot displays 208,506 individual cells, grouped by normal and tumor tissues, and categorized by major cell lineages. (B) The relative proportion of the eight cell subsets from cancer or normal tissues from lungs. nlung, normal tissues from lungs; tlung, cancer tissue from lungs. (C) Boxplot showing the scores of cholesterol metabolism gene sets by tissue origin and cell subsets. ∗∗∗∗, *p* < 0.0001. (D) t-SNE plot colored by the scores of cholesterol metabolism gene sets in tlung. (E) t-SNE plot of myeloid cells, color coded by clusters and cell subsets as indicated. (F) The relative proportion of the 11 cell subsets from cancer and normal tissues from lungs. (G and H) Boxplot showing the scores of cholesterol metabolism and TNF signaling pathway gene sets by tissue origin and cell subsets in myeloid cell lineage. nlung, normal tissues from lungs; tlung, cancer tissue from lungs. ∗∗*p* < 0.01; ∗∗∗∗*p* < 0.0001; ns, not significance.

A recent study has suggested the potential role of lipid metabolism in the prognosis of LUAD patients,^23^ which led us to further incorporate scRNA-seq data to understand the role of lipid metabolism in LUAD. We included the datasets GSE131907 and GSE153935 containing LUAD and normal tissues for further analysis, as discovery data and validation data, respectively. In the GSE131907, 8 cell lineages were obtained after clustering and annotation (Figures 5A and 5B). Then, we used AUCell to calculate the gene set score of each cell type to compare the lipid metabolism characteristics of different cells. Interestingly, we found that myeloid cells had significantly the highest scores of lipid and atherosclerosis (Figures S8A and S8B). Importantly, cholesterol metabolism was also scored high in myeloid cells (Figures 5C and 5D).

To further elucidate the subpopulations of myeloid cells and their lipid and cholesterol metabolism, subclustering of the myeloid cells was performed as previously described (Figures 5E and 5F). Alveolar mononuclear macrophages are mainly present in lung tissues, while LUAD tissues exhibit a significant enrichment of monocyte-derived macrophages ([mo-Macs] anti-inflammatory and pro-inflammatory types). Among myeloid cell subtypes, we found that the cholesterol metabolism score of mo-Mac was significantly higher in LUAD, while its tumor necrosis factor (TNF) signaling pathway score was markedly reduced (Figures 5G and 5H).

In the validation cohort GSE153935, the results showed that myeloid cells had the highest lipid and atherosclerosis and cholesterol metabolism scores, consistent with the conclusions in GSE131907 (Figures S8C–S8E).

### Remodeling of cholesterol metabolism that promotes M2-subtype polarization in mo-Macs from LUAD

**Figure 6:**
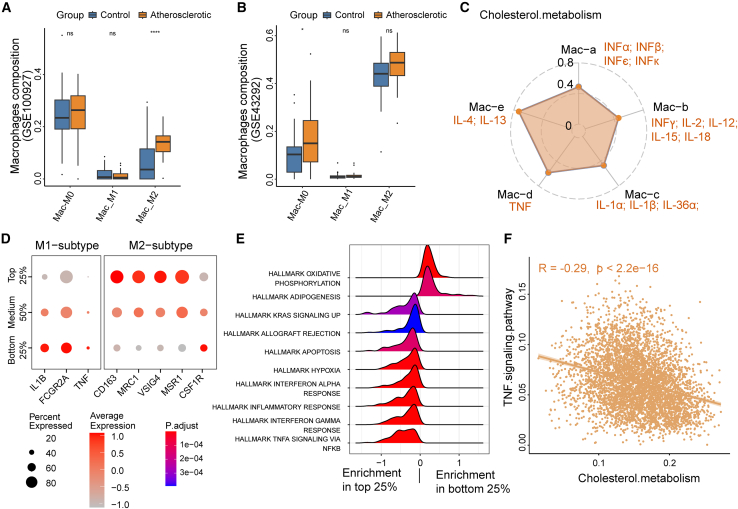
Remodeling of cholesterol metabolism that promotes M2-like macrophage polarization in mo-Mac from LUAD (A and B) Boxplots illustrating the fractions of macrophage subtypes in the atherosclerotic group and control group based on RNA-seq data from GSE100927 (A) and GSE43292 dataset (B). ∗*p* < 0.05; ∗∗∗∗*p* < 0.0001; ns, not significance. (C) IREA analysis shows an enrichment of macrophage polarization state between the top 25% and bottom 25% cell subpopulations based on the cholesterol metabolism scores in mo-Mac. (D) The expression of markers of M1-subtype and M2-subtype macrophage between the top 25%, the bottom 25%, and the remaining 50% subpopulations based on the cholesterol metabolism scores. (E) Pathways significantly enriched in the top 25% compared with the bottom 25% subpopulations in mo-Mac. (F) Scatterplot shows the relationship between the level of cholesterol metabolism and that of the TNF signaling pathway.

AS is the basis of numerous cardiovascular diseases, in which macrophages form foam cells to form unstable atherosclerotic plaques, which play a central role in the initiation and development stages of atherosclerotic pathogenesis.^24^^,^^25^ To investigate the role of macrophages in AS, we analyzed the bulk RNA-seq data of human-derived atherosclerosis from carotid, femoral, and infra-popliteal territories (GSE100927), and we found that macrophages have the highest proportion among immune cells. Compared with normal arteries, the M2 level of arteriosclerotic tissue was significantly higher, while the M0 and M1 levels were not significantly different (Figure 6A). Although insignificant, this trend was confirmed in another dataset (GSE43292) (Figure 6B).

The macrophage polarization into an anti-inflammatory M2 state is widely regarded as having a beneficial effect against AS.^26^ Meanwhile, M2 macrophages secrete anti-inflammatory cytokines such as interleukin (IL)-10 to elicit anti-inflammatory responses, participate in tumor angiogenesis and extracellular matrix remodeling, and can also control the inflammatory response by downregulating the functions mediated by M1 macrophages, thereby promoting tumor progression.^27^^,^^28^ To investigate the effects of metabolism on cellular functions, we first selected the top 25% and bottom 25% cell subsets in mo-Mac from LUAD for differential expression analysis based on the shared pathway scores. Then, we conducted the IREA on the differentially expressed genes. The results show strong enrichment of macrophage polarization toward an IL-4/-6-induced polarization state in higher cholesterol metabolism (Figure 6C). By grouping mo-Mac based on their cholesterol metabolism scores, we found that markers related to the M1 subtype (IL1B, FCRG2A, and TNF) were mainly expressed in the bottom 25% subpopulations, while markers related to the M2 subtype (CD163, MRC1, VSIG4, and MSR1) were mainly expressed in the top 25% subpopulations (Figure 6D). Further pathway enrichment analysis results suggested that the interferon gamma response and TNFA signaling via nuclear factor-κB pathways were enriched in the bottom 25% mo-Mac (Figure 6E). Additionally, we found that the level of cholesterol metabolism was negatively correlated with the level of the TNF signaling pathway (Figure 6F). In summary, our results suggest macrophage polarization into an anti-inflammatory M2 state against atherosclerosis, which may play an important role in the occurrence and development of LUAD and AS.

### Cardiometabolic medications for LC-CMTs

Given that cardiometabolic disorders frequently occur together, we utilized the shared genes identified by the four methods to pinpoint medications for addressing LC in the presence of comorbidities. We explored three large drug-interaction databases (DrugCentral, DGIdb, and PharmGKB) to identify drugs that interact with any of the 731 candidate genes, resulting in 171 drugs. Then, we restricted the drugs approved by the Food and Drug Administration and those that were already utilized to treat cancer or cardiometabolic disorders, which reduced the list to 87 drugs that target 53 genes. Collectively, 87 candidate drugs divided into 6 categories were investigated, namely, antineoplastic drugs (37 drugs), antihypertensive drugs (30 drugs), antiarrhythmic drugs (6 drugs), glucose-lowering drugs (6 drugs), lipid-lowering drugs (3 drugs), and other antimetabolic drugs (6 drugs). These drugs have largely been sanctioned for the treatment of a range of cancers or CMDs.

**Figure 7:**
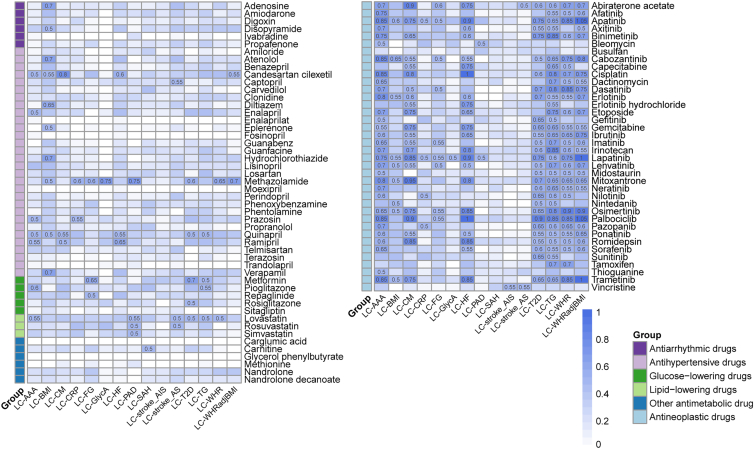
Matching between disease pathological pathways, inferred from shared genes for each trait pair, and drug pharmacological pathways Pairing scores with values not less than 0.5 are marked in the squares.

In each trait pair, one drug has one target in most situations (Figure S9A). For some drugs, there were more than 2 targets, especially in antineoplastic drugs. In our study, we focus on the cardiometabolic drug in more detail, and the gene could be targetable across the five category drugs, which makes it more potential to become a target for comorbid diseases (Figure S9B). When pairing scores were more than 0.5, the best-matching medications were defined (Figure 7). Antineoplastic drugs affect metabolic diseases and traits, which are often associated with side effects. For antihypertensive drugs, candesartan cilexetil, methazolamide, and ramipril were suggested for various trait pairs, including LC-CM, LC-HF, LC-AAA, and LC-PAD. Metformin obtained high pairing scores in LC comorbid with CMTs, such as fasting glucose, TGs, and T2D, while lovastatin and rosuvastatin obtained high pairing scores in LC comorbid with CMDs, such as AAA, PAD, AS, T2D, TGs, and WHR. These findings supported their extended application in treating comorbid conditions in LC.

## Discussion

Using summary-level GWAS data, this study delineated the shared genetic mechanisms of LC and CMTs by investigating genetic correlations, pleiotropic loci and genes, as well as pathways. Additionally, analyzing bulk and scRNA-seq identified the effects of cholesterol metabolism on M2-subtype macrophage polarization in mo-Mac, which may play an important role in the occurrence and development of LUAD and AS.

Early detection of LC improves treatment effectiveness and patient survival, which makes screening in high-risk groups especially important, but screening compliance is frequently low.^29^ In a retrospective study by Bintein et al., it was found that patients with atherosclerotic PAD constitute a high-risk population for LC, with about 3% of patients with PAD being diagnosed with LC.^30^ In a secondary analysis of the Aspirin in Reducing Events in the Elderly (ASPREE) trial, Muhandiramge et al. found an increased incidence of cardiovascular disease (including myocardial infarction, HF, and ischemic stroke) among older adults with LC.^31^ Although cardiovascular disease and stroke follow LC or its treatment, shared risk factors, such as obesity and smoking, also contribute to these comorbidities.^32^ Our study has found that LC was significantly related to CMDs, such as PAD and HF. While observational studies show an inverse association between BMI and LC, our MR analysis revealed a positive relationship (OR [95% CI] = 1.25 [1.16–1.34]), which remained consistent across multiple sensitivity analyses and aligns with previous MR analysis.^33^ This difference highlights several MR advantages: genetic instruments reflect lifelong BMI exposure and MR minimizes confounding (especially from smoking), eliminates reverse causation, and may provide more accurate causal estimates. These contradictory findings suggest the BMI-LC relationship is more complex than previously assumed, with MR potentially revealing biological mechanisms masked by confounding in observational studies, underscoring the need for further investigation. Based on the above findings, it may be recommended that patients with obesity, HF, or PAD be considered high-risk individuals for LC screening.

Larger sample sizes correspond to higher statistical power when studying shared SNPs and genes, uncovering not only known genetic variants associated with LC but also novel loci. The variants in HLA, CHRNA5-CHRNA3-CHRNB4, CYP2A6, and PSMA4 reported to be related to lung function^34^ were suggested as risk variants associated with traits paired with LC. There were the most shared pleiotropic loci between LC and PAD, which could explain the comorbidity of PAD and LC.^30^ Cardiovascular risk marker ADAMTS7^35^ was a newly discovered risk gene of LC, and there were shared variants including rs7166723, rs77719127, and rs10894196 in LC-AAA. The dysregulation of the immune response or metabolism was a feature of LC^36^; genetic variants were found in the immunometabolism gene AK5, which were the shared genetic loci between LC and BMI.

By constructing LC-CMTs shared functional gene sets and scoring through AUCell, we conduct further exploration to discover the co-pathogenesis between LC and CMTs. A recent study found that abnormal lipid metabolism in lung adenocarcinoma was associated with poorer patient outcomes and contributed to the remodeling of the tumor immune microenvironment (TIME).^23^ Interestingly, the scores of atherosclerosis lipid and cholesterol metabolism were high in myeloid cells, suggesting that they may play an important role in LC. In addition, macrophages were the most enriched cell by CSEA analysis, including macrophages from blood, liver and lung, suggesting them as hubs for LC and CMTs.

AS is the underlying basis for various CMDs, including CAD, HF, PAD, AA, arrhythmias, stroke, and some metabolic syndromes.^17^ Inflammatory features or signaling pathways observed in endothelial cells in atherosclerotic regions, such as COX-2 and the LOX-1/ox-LDL pathway, have also been observed in tumor endothelial cells.^37^ Considering that macrophages form foam cells to form unstable atherosclerotic plaques, which play a central role in the initiation and development stages of AS pathogenesis,^24^^,^^25^ we further explored the common roles played by macrophages in atherosclerotic diseases and lung adenocarcinoma. Through the microenvironment deconvolution analysis of GSE100927 and GSE100927, M2 macrophages were found to be more abundant in sclerotic plaques than in normal vascular tissue, which is associated with resistance to disease progression.^38^ Furthermore, IREA suggested that macrophages polarized toward an IL-4/-13-induced polarization state in higher cholesterol metabolism. IL-4 and IL-13 drive a unique “Mac-e” state that is characterized by immunosuppressive genes and reparative M2-like states and related to enhancing tumor cell proliferation and suppressing CD8^+^ T cell activation.^39^ TNF signaling is a key pathway responsible for activating macrophage-derived CXCL10 and CXCR3^+^ cytotoxic T lymphocytes and is related to the inflamed TIME.^40^ Consistent with the M2-like-induced immunosuppressive microenvironment, we found that the cholesterol metabolism score was significantly negatively correlated with the TNF signaling pathway score.

The statin family is well-known for its anti-atherosclerotic effects, and in our study, rosuvastatin and lovastatin were identified as potential drugs for LC and cardiovascular diseases. The mechanism by which statins exert anti-tumor effects through the regulation of macrophage polarization^41^ aligns with the findings of our study. It was found that simvastatin repolarized tumor-associated macrophages (TAMs) by regulating the cholesterol-related LXR/ABCA1 pathway, promoting the phenotypic shift of TAMs from the M2 type to the M1 type.^42^ And this polarization increased the levels of TNF-α while inhibiting the expression of transforming growth factor-β, thereby remodeling the tumor microenvironment. In a cohort study involving 262 surgically resected primary human lung adenocarcinomas, Dujaily et al. reported that statins exhibited a dose-dependent effect in reducing the presence of protumorigenic TAMs, characterized by the markers CD68^+^ CD163+, within both the tumor stroma and parenchyma.^43^ In our study, macrophages were the most enriched cell, including macrophages from the blood, liver, and lung by CSEA, suggesting them as hubs for LC and CMTs. By analyzing scRNA-seq data from LUAD and normal tissue, we also found that there was enrichment of macrophage polarization toward an M2-like state in higher cholesterol metabolism. This connection provides theoretical support for further exploring the potential role of statins in tumor immunotherapy.

### Limitations of the study

There were several strengths in our study. First, we incorporated GWAS summary statistics for LC and 36 CMTs to comprehensively investigate shared genetic associations among these comorbidities. Second, cross-trait meta-analysis enhances statistical power by increasing sample size, facilitating the identification of both known and novel genetic variants and loci associated with LC-CMTs. Third, integrating GWASs data with sequencing analysis allowed us to further explore the shared pathogenic mechanisms between LC and CMTs. However, there were also some limitations. First, this study primarily focused on the genetic mechanisms shared by traits, rather than modifiable lifestyle factors like smoking and sedentary behavior. This focus arises from the observation that human traits and diseases generally exhibit greater heritability compared to lifestyle and environmental exposures, while it is important to acknowledge that other elements can significantly contribute as well. Second, CMTs cover a variety of diseases and physiological characteristics. The 36 CMTs included in our study are only representative, and future studies need to include more diseases and traits. Third, considering that AS is the underlying basis for various CMTs, the bulk and scRNA-seq data from atherosclerotic plaque and LC were included to find the common biologies, rather than all other features. With increasing data and improved methods, we will be able to further explore the physiological mechanisms of the comorbidity of LC and CMTs, which will provide therapeutic insights for treating patients with comorbidities in the clinic.

In summary, our research uncovered significant genetic links and identified common risk loci shared between LC and CMTs, emphasizing the potential involvement of macrophage cholesterol metabolism. Notably, we find that macrophage polarization into an anti-inflammatory M2 state against atherosclerosis may play an important role in the occurrence and development of LUAD. These findings provide valuable insights into the shared genetic architecture between LC and CMTs, contributing to a deeper understanding of their pathogenesis and therapeutic strategies.

## Resource availability

### Lead contact

Further information and requests for resources should be directed to and will be fulfilled by the lead contact, Ping He (hp5567@163.com).

### Materials availability

This study did not generate new unique reagents.

### Data and code availability

• Data: This study did not generate any new data. A comprehensive list of all publicly available datasets used in this study is provided in the STAR Methods and the key resources table.
• Code: All code utilized in this study is publicly available and is described in the STAR Methods and the key resources table.
• Any additional information required to reanalyze the data reported in this article is available from the lead contact upon request.

## Acknowledgments

This study was funding by the Major Project of Guangzhou National Laboratory (GZNL2023A03001), the Research Projects of 10.13039/100009659Guangzhou Medical University (2024SRP071), the 10.13039/501100001809National Natural Science Foundation of China (82473364, 82172811, and 82260546), the 10.13039/501100004479Natural Science Foundation of Jiangxi Province (20242BAB25473, 20224BAB206062, and 20224BAB216043), the Science and Technology Project of 10.13039/501100009102Education Department of Jiangxi Province (GJJ2201426), the Ganzhou City Science and Technology Plan Project (GZ2023ZSF096), and the Ganzhou City Health Commission municipal scientific research project (2023-2-083, GZWJW202402275).

## Author contributions

P.H., Y.X., and Z.Q. were responsible for the conceptualization, methodology, and supervision of the project. Q.L., X.-Y.W., and E.C. contributed to the methodology, software programming, detailed analysis, visual representation, initial draft preparation, and extensive manuscript revisions. Additionally, Q.L., X.-Y.W., and C.S. conducted a thorough review and refinement of the manuscript. All authors played significant roles in the publication process and endorsed the final version of the manuscript.

## Declaration of interests

The authors declare no competing interests.

## Declaration of generative AI and AI-assisted technologies in the writing process

During the preparation of this work, the authors used Claude in order to improve language and readability. After using this tool, the authors reviewed and edited the content as needed and take full responsibility for the content of the published article.

## STAR★Methods

### Key resources table

**Table:** 

REAGENT or RESOURCE	SOURCE	IDENTIFIER
**Deposited Data**		

GWAS summary statistics of LC	McKay et al.^44^	GWAS Catalog, GCST004748
GWAS summary statistics of LC (replication)	FinnGen R12^45^	C3_BRONCHUS_LUNG_EXALLC
scRNA-seq of LUAD	Kim et al.^46^	GEO: GSE131907
scRNA-seq of LUAD (validation)	Hanley et al.^47^	GEO: GSE153935
RNA-seq of Atherosclerotic plaque	Steenman et al.^48^	GEO: GSE100927
RNA-seq of Atherosclerotic plaque (validation)	Ayari et al.^49^	GEO: GSE43292

**Software and Algorithms**		

R (version 4.1.0)	The R Project for Statistical Computing	R Core Team
LDSC	Bulik-Sullivan et al.^50^	https://github.com/bulik/ldsc
TwoSampleMR	Hemani et al.^51^	https://github.com/MRCIEU/TwoSampleMR
LCV	O'Connor et al.^52^	https://github.com/lukejoconnor/LCV
DrugtargetMR	Lyu et al.^53^	https://github.com/qzq1111/DrugtargetMR-workflows
MTAG	Turley et al.^54^	https://github.com/omeed-maghzian/mtag
CPASSOC	Li et al.^55^	https://pmc.ncbi.nlm.nih.gov/articles/PMC6417431/
ANNOVAR	Wang et al.^56^	https://annovar.openbioinformatics.org/
Coloc	Giambartolomei et al.^57^	https://github.com/chr1swallace/coloc
SMR	Zhu et al.^58^	https://cnsgenomics.com/software/smr/
FUSION-TWAS	Gusev et al.^59^	http://gusevlab.org/projects/fusion/
MAGMA	de Leeuw et al.^60^	https://ctg.cncr.nl/software/magma
GCTA-fastBAT	Bakshi et al.^61^	https://yanglab.westlake.edu.cn/software/gcta/
deTS	Pei et al.^62^	https://github.com/broadinstitute/gtex-pipeline/tree/master/deTS
WebCSEA	Dai et al.^63^	https://bioinfo.uth.edu/webcsea/
IOBR	Zeng et al.^64^	https://github.com/IOBR/IOBR
Seurat	Hao et al.^65^	https://satijalab.org/seurat/
AUCell	Aibar et al.^66^	https://github.com/aertslab/AUCell
IREA	Cui et al.^39^; Cao et al.^67^	https://www.immune-dictionary.org/app/home
clusterProfiler	Wu et al.^68^	https://guangchuangyu.github.io/software/clusterProfiler

### Method details

#### GWAS summary statistics collection

By combining the OncoArray results with previously reported LC GWAS, McKay et al. have employed fixed-effects models to analyze 29,266 cases and 56,450 controls of European ancestry, and the corresponding GWAS statistics were obtained from the GWAS Catalog under ID GCST004748.^44^ GWAS statistics for LC subtypes, including lung adenocarcinoma (LUAD), lung squamous cell carcinoma (LUSC), and small cell lung cancer (SCLC), were included for subtype-specific analyses, while another GWAS summary data of LC downloaded from FinnGen R12 was used for replication analyses.^45^ The details about the GWAS data for CMTs, including diagnosis, recruitment procedures, and quality assurance measures, are available in the original research. Given that this study’s analysis did not include personally identifiable information, no further ethics committee permission was needed.

Table S1 compiles all of the GWAS statistics, including 36 GWAS statistics about CMT, the data for which were gathered from large consortia including FinnGen, UK Biobank, etc. Concerning type 2 diabetes, the DIAMANTE consortium provided the GWAS statistics for 180,834 cases and 1,159,055 controls (51.1% European descent).^69^ For the summary statistics for blood glucose, up to 281,416 individuals without diabetes (70% European ancestry) were included in the *trans*-ancestral meta-analysis, and the ancestry-specific summary-level results were available through the MAGIC website (https://www.magicinvestigators.org/).^70^ For GWAS summary data for lipids, the European-specific GWAS meta-analysis results were available at: http://csg.sph.umich.edu/willer/public/glgc-lipids2021.^71^

#### Quality control of the GWAS summary statistics

To ensure the robustness and accuracy of GWAS summary statistics analysis, we undertook multiple quality assurance steps with reference to previous studies.^72^ First, we standardized all summary statistics to the GRCh37 human genome assembly, converting variants from GRCh38 using liftover when necessary. These unified genomic coordinates enabled us to utilize reference data from build 37 for all downstream analyses. Subsequently, we filtered variants to include only those present in the 1000 Genomes Project Phase 3 European reference panel with a minor allele frequency (MAF) greater than 1%, thereby excluding rare variants that could introduce noise. We retained only variants with reliable rsIDs and clear single-nucleotide alleles and removed duplicates to ensure consistent identification across datasets. We also checked for allele flipping to ensure consistent directionality of allelic effects and frequencies across datasets. For each analytical method, such as the LDSC analysis, we formatted the standardized files to meet the input requirements of LDSC, allowing direct use without pre-processing. Finally, the rigorously quality-controlled GWAS data were prepared for subsequent genetic analyses to yield robust results.

#### Genome-wide association analysis

Linkage Disequilibrium Score Regression (LDSC) was employed to calculate the genome-wide association analysis for 36 pairwise traits.^50^ It assesses the individual impacts of polygenic effects by analyzing the correlation between linkage disequilibrium scores, and produces genetic correlations by evaluating deviations from the null hypothesis through chi-squared values. To adjust for sample overlap among the studies, LDSC also reports a self-reported intercept in the analysis.

Complete negative genetic correlation is represented by a value of −1, and complete positive genetic correlation is represented by a value of 1. For the LDSC analysis of 36 trait pairs, the *p*-values were adjusted using the classic Benjamini-Hochberg false discovery rate (FDR) procedure to control false positives, with the statistically significant threshold set at an adjusted *p*-value (Padj) < 0.05.

#### Bidirectional causal relationships

We used bidirectional Mendelian randomization (MR) analysis to determine the causal associations among the LC-CMTs trait pair. Three assumptions need to be satisfied for a genetic variant to be a valid instrumental variable (IV) in MR analysis: (1) The variant is associated with the exposure. (2) The variant is not associated with the outcome through a confounding pathway. (3) The variant has no direct impact on the outcome, but may only have an indirect effect via the exposure. Following these criteria, when exploring the causal relationship from CMTs to LC, we selected SNPs associated with the exposure (*p* < 5 × 10−8, r2 < 0.001, kb = 10,000) based on European reference data, and excluded those directly associated with LC or smoking for MR analysis. We conducted the inverse-variance weighted method (IVW) as the main analysis by the “TwoSampleMR” package in R.^51^ Another four MR methods were used as sensitivity analyses, including weighted mode, simple mode, weighted median, and MR-egger. If the *p*-value of IVW still met the significance after FDR correction (*n* = 72) and the other four analysis methods showed consistent trends, it suggested the existence of a robust causal effect on exposure and outcome.

Furthermore, we inferred putative causal relationships between LC and CMTs using the LCV (latent causal variable) model.^52^ The LCV model assumes that the genetic correlation between two traits is mediated by a latent variable, which can be quantified by calculating the genetic causality proportion (GCP). The GCP value ranges from −1 to 1, with higher absolute values suggesting more substantial partial causality, such as 0 for no causal relationship and 1 for a complete causal association.

#### Cross-trait meta-analysis and pleiotropic loci

The paired traits that met significance in LDSC analysis were considered for additional cross-trait meta-analyses with the DrugtargetMR package to uncover the shared pleiotropic variant.^53^ Before this analysis, we summarized the sample sizes of the GWAS studies and the number of valid shared SNPs for each trait pair available for cross-trait meta-analysis to ensure the reliability of the results. For the first used one, the multi-trait analysis of genome-wide association studies (MTAG) is a method that utilizes generalized inverse-variance-weighted meta-analysis to simultaneously examine multiple traits, while also accounting for potential sample overlap between GWAS.^54^ MTAG provides increased statistical strength and reduces the inflation of the false discovery rate for trait pairs, particularly when there are strong correlations. Furthermore, the outcomes derived from MTAG remain robust, even in the presence of sample overlap within investigated traits. The second method we conducted was the cross-phenotype association test (CPASSOC), which combines various traits to identify shared variants while accounting for demographic structure and hidden relatedness.^55^ SHet, an enhanced version of SHom, allows for heterogeneous effects arising from different phenotypes and studies. We performed the SHom and Shet analysis and compared their results to assess potential heterogeneity of effects between traits.

The significant pleiotropic SNPs were defined as variants that meet the criteria of *p*_CPASSOC_ < 5 × 10^−8^, *p*_MTAG_ < 5 × 10^−8^, and *p*_single-trait_ < 1 × 10^−3^ for both traits.^55^ And linkage disequilibrium (LD) clumping was conducted for to identify independent SNPs with the following parameters: p1 was set to 5e-8, p2 to 1e-5, r2 to 0.2, and kb to 500. Additionally, we implemented more stringent clumping thresholds to further confirm independent SNPs significantly associated with LC-CMTs trait pairs. Next, we functionally annotated the shared variations using ANNOVAR.^56^

#### Colocalization analysis

The coloc R package was used to determine whether there were shared risk loci for LC and CMTs.^57^ For each of the shared SNVs in LC-trait, the variants located within 500 Kb of the index SNP were extracted to assess the likelihood that both traits share a common causal variant (PPH4). Loci exhibiting a probability exceeding 0.7 were significant according to ref. ^73^^,^^74^.

#### Gene-based association analysis

Given that significant pleiotropic SNP functional annotations in ANNOVAR are solely based on physical proximity, we further employed four gene-based approaches to identify significant shared genes for LC and CMTs: SMR,^58^^,^^75^ FUSION-TWAS,^59^ MAGMA,^60^ and GCTA-fastBAT.^61^ For all four gene-level analyses, the input files came from meta-analysis using MTAG. In each method, the significance threshold of *p*-values was adjusted using FDR correction, with the statistically significant threshold set at Padj <0.01.

Summary-data-based Mendelian Randomization (SMR) analysis combines statistics for GWAS and expression quantitative trait locus(eQTL) studies to identify genes that show associations between their expression levels and complex traits, either due to pleiotropic effects or causal relationships.^58^^,^^75^ There are three possible explanations for a significant SMR association: linkage, where there are multiple causal variants for both gene expression and disease, pleiotropy, where the causal variant has pleiotropic influences with both gene expression and disease, or causal effect, where the causal variant influences disease risk through alterations in gene expression. The HEIDI-outlier test is used in the SMR method to differentiate between linkage and pleiotropy. In this study, SMR was performed employing *cis*-eQTL summary data from GTEx V8 for whole blood and relevant tissues.^76^ To balance candidate gene identification and false positive control, several studies excluded significant SMR associations due to LD using P_HEIDI_ < 0.01,^73^^,^^77^^,^^78^^,^^79^ a criterion also adopted in this study. Specifically, genes with FDR-adjusted *p*-value (Padj) < 0.01 and P_HEIDI_ > 0.01 were defined as significant. Functional Summary-based Imputation (FUSION-TWAS) is a powerful toolkit for conducting transcriptome-wide association studies.^59^ It leverages precomputed gene expression weights and GWAS summary statistics to estimate the association between each gene and a specific disease. To address the potential low power of TWAS when using QTL data with small sample sizes, we conducted TWAS analysis using the top 3 weights built from sparse canonical correlation analysis (sCCA), which integrates eQTL data across multiple tissues from the Genotype-Tissue Expression (GTEx) project.^80^ Then, we employed the aggregate Cauchy association test (ACAT) to identify genes associated with the comorbid diseases (sCCA-TWAS+ACAT approach). The corresponding code for sCCA-TWAS + ACAT can be found in the GitHub repository (https://github.com/fenghelian/sCCA-ACAT_TWAS). By utilizing this extensive resource, we were able to investigate cross-tissue gene-trait associations, thus obtaining a better comprehension of the underlying mechanisms.

SMR and FUSION-TWAS utilize eQTL to perform analyses, while MAGMA and GCTA-fastBAT primarily rely on proximity for gene burden tests. MAGMA (Multi-marker Analysis of GenoMic Annotation) is a rapid and adaptable approach for analyzing genes and gene sets within GWAS statistics.^60^ On the other hand, GCTA-fastBAT offers an efficient set-based analysis for complicated traits utilizing statistics of GWAS and the data of LD from a reference population.^61^ Using the European ancestry as the LD reference, we performed MAGMA and GCTA-fastBAT with default settings.

#### Gene set enrichment analysis

To investigate the biological pathways of these shared genes reported by the four methods, we performed gene set enrichment analysis, which drew from the Kyoto Encyclopedia of Genes and Genomes (KEGG), as well as the Gene Ontology (GO) databases.^68^ For multiple tests, pathways with the FDR-adjusted *p*-value <0.01 were defined as significant.

#### Tissues and cell types enrichment analysis

The shared genes for LC and CMTs were identified as those meeting the statistically significant threshold of FDR-adjusted *p*-value <0.01 in any of four gene-level analysis methods. Many genetic variants lead to diseases or traits by modulating tissue-specific regulation.^81^ Pei et al. analyzed expression data from 47 tissues in the GTEx project to quantify tissue specificity for each gene, including 14,725 protein-coding, non-housekeeping genes, and subsequently conducted tissue-specific expression analysis (TSEA) using Fisher’s exact test or *t* test to identify TSG expression and regulation. In our study, we utilized the deTS R package with the built-in GTEx reference panels to identify the most relevant tissues for the shared genes among LC-trait pairs using Fisher’s exact test.^62^ To account for multiple testing, the FDR-corrected *p*-value <0.05 was considered significant.

Additionally, we utilized WebCSEA to conduct the CSEA (cell-type-specific enrichment analysis) with the shared genes among LC-trait pairs. This analysis allows for querying gene sets against tissue-cell-specific expression profiles derived from a total of 111 scRNA-seq panels of human tissues and 1,355 tissue-cell types from 61 different general tissues across 11 human organ systems.^63^ Consistent with the original developers' criteria, genes were identified as cell type-specific if they ranked within the top 5% of t-statistic scores for the respective cell type. We selected 353 cells from tissues associated with LC-CMTs (heart, blood, artery, lung, pancreas, liver, kidney, and adipose tissue) for CSEA, and then conducted Fisher’s exact test to assess whether the shared genes were cell-type-specific.

#### Microenvironment deconvolution analysis for bulk RNA-seq data

Considering the importance of immune cells in comorbidities, we utilized bulk RNA sequencing data of atherosclerotic plaques and normal arteries to calculate the immune cell infiltration scores using the deconvo_tme function with the CIBERSORT algorithm via the IOBR package.^64^ The first dataset of 69 human-derived atherosclerotic and 35 healthy arteries was downloaded from GSE100927,^48^ and another GSE43292,^49^ which comprised 32 paired atheroma plaque and macroscopically intact tissue, was used as a validation.

#### Single-cell RNA sequencing data analysis

To elucidate the cell heterogeneity of LC-CMTs shared pathways in LC, we obtained single-cell RNA sequencing data of 11 paired normal and lung adenocarcinoma (LUAD) from GSE131907 as a discovery dataset.^46^ Another dataset comprised of human control (*n* = 6) and LC (*n* = 12, seven squamous cell carcinomas, and five adenocarcinomas) samples was downloaded from GSE153935 as a validation cohort.^47^ The quality control, cell clustering, and annotation were analyzed with Seurat software (4.3.0.1) following the online guide (https://satijalab.org/seurat/).^65^ Unsupervised clustering of the cells was performed based on the gene expression profiles using the FindClusters function with the Louvain algorithm, and the clusters were visualized using t-distributed Stochastic Neighbor Embedding (tSNE). Subsequently, cell types were annotated according to the expression of canonical markers, including multiple distinct mesenchymal, immune and epithelial cells.

Subsequently, the AUCell R package was used to assess the activity of shared pathways in individual cells.^66^ To mitigate the effect of cells with more reads (due to technical or biological reasons) having higher average scores for most gene sets, we utilized the gene expression profile normalized by the LogNormalize method in the NormalizeData function of Seurat software to derive gene expression rankings for each cell. Then, we applied the LC-CMTs shared functional gene sets to evaluate each cell’s scores based on these rankings, where the AUC values indicate the proportion of genes that belong to the top-ranking category defined within the pathway.

The Immune Response Enrichment Analysis (IREA) was used to infer cytokine activity and immune cell polarisation during immunization.^39^^,^^67^ In our study, it was used to impute cell polarisation during the level of lipid-related metabolism varied based on the differentially expressed genes (DEGs) in the top 25% and bottom 25% cell subpopulations based on pathway activity scores.

#### Cardiometabolic medications for LC-CMTs

To determine which medications best fit the comorbidity, we used the clusterProfiler R package to calculate the enriched pathological pathways for each LC-trait pair.^68^ Following that, we took several steps to identify potential drugs: (1) searched three well-established pharmacological databases (DrugCentral,^82^ DGIdb,^83^ and PharmGKB^84^) to identify medications targeting our identified shared genes. These comprehensive resources offer extensively curated information on drug-gene interactions and pharmacological relationships, collectively serving as invaluable tools for drug discovery, repurposing strategies, and personalized medicine applications. (2) restricted the compounds approved by the FDA and those that were already utilized to treat cancer or cardiometabolic disorders. Next, we required all affected genes in the three datasets, and used clusterProfiler to calculate the enriched pharmacological pathways of the nominated compounds respectively. Lastly, we calculated the scores between the drug’s pharmacological pathways and the trait-pair’s pathological pathways.^85^ Briefly, this score quantifies overlap between pathological and pharmacological pathways by matching their top enriched terms across two tiers: Tier 1 (top 20 terms, weight = 1.0) and Tier 2 (terms 21–50, weight = 0.5). The pairing score is calculated as the sum of three components: the weighted matches in mutual terms from Tier 1, and the weighted matches from both pathology and pharmacology terms in Tier 2, all normalized by the number of Tier 1 terms.

### Quantification and statistical analysis

Data analysis and visualization were conducted using R software (version 4.2.2). For comparisons between two groups, a two-tailed Student’s *t* test was employed when data conformed to the assumption of normality; otherwise, the non-parametric Wilcoxon rank-sum test was used. For comparisons across three or more groups, one-way analysis of variance (ANOVA) was performed on normally distributed data. Statistical significance was denoted by asterisks as follows: ∗*p* < 0.05, ∗∗*p* < 0.01, ∗∗∗*p* < 0.001, and ∗∗∗∗*p* < 0.0001. Unless specified otherwise, all tests were two-tailed.

## Supplemental information

Document S1. Figures S1–S9

Table S1. Summary of GWAS data

Table S2. Results of LDSC genetic correlation analysis for 36 LC-CMTs trait pairs

Table S3. The results of bidirectional MR analysis between LC and CMTs

Table S4. The results of LCV model between LC and CMTs

Table S5. Replication analysis, the results of bidirectional MR analysis of LC (FinnGen R12) and CMTs

Table S6. The results of bidirectional MR analysis between LC subtypes and CMTs

Table S7. Cross-trait meta-analysis between LC and CMTs with different clumping parameters

Table S8. The list of LC-trait pairs-related genes identified by four gene-based analyses
