# Supplementary material for: Multi-trait association analysis reveals shared genetic architecture between lung cancer and cardiometabolic diseases
Source: iScience. 2025 Nov 19;28(12):114129. doi: 10.1016/j.isci.2025.114129 (PMC12744264; doi:10.1016/j.isci.2025.114129)
Supplement: Document S1. Figures S1–S9 [file mmc1.pdf]

iScience, Volume *28*

Supplemental information

Multi-trait association analysis reveals shared genetic architecture between lung cancer and cardiometabolic diseases

Qiong Lyu, Xuan-Yu Wang, Erhong Chen, Chanjuan Sun, Zhengang Qiu, Yingyu Xie, and Ping He

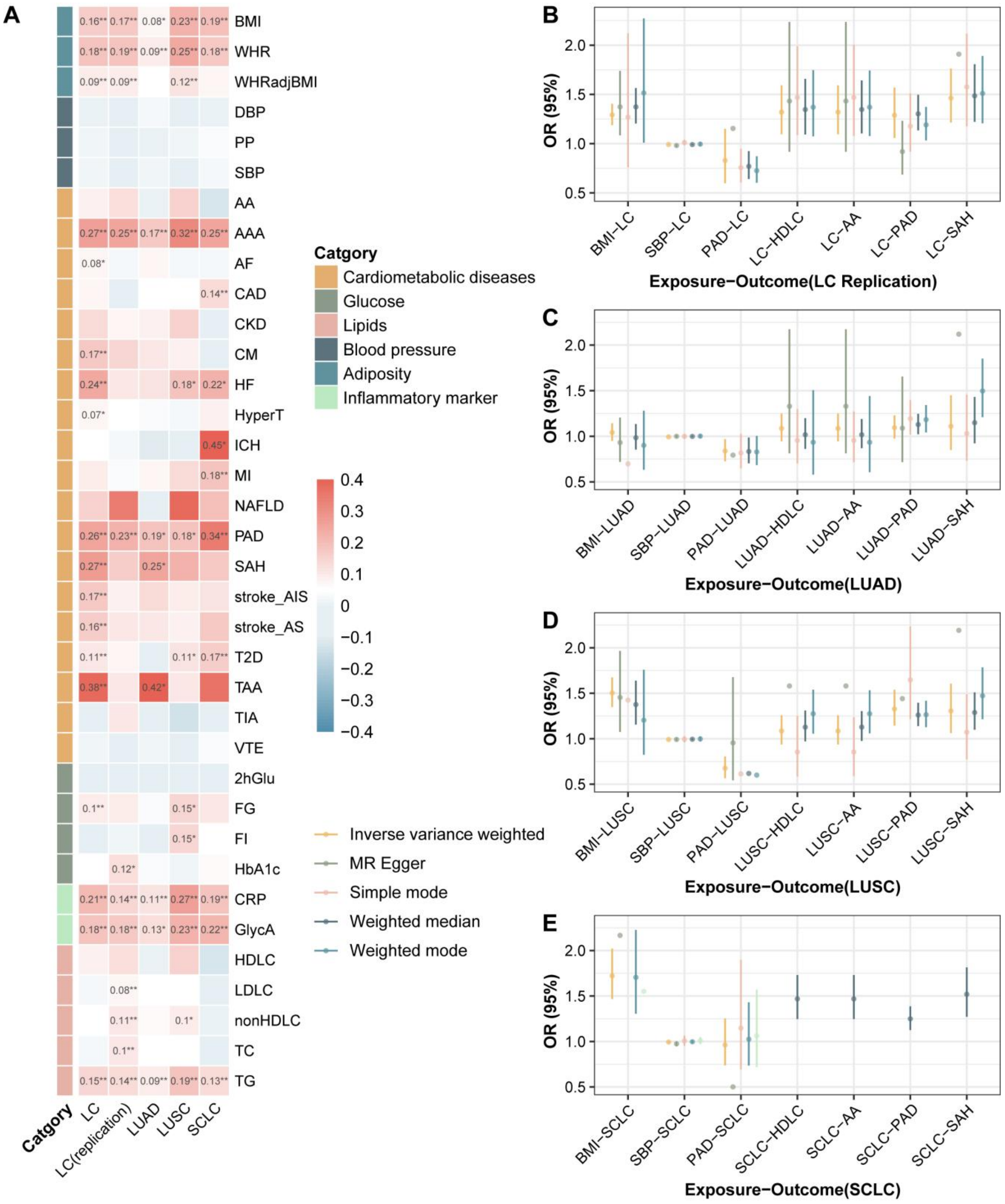

Supplementary Figure 1. The comprehensive landscape of genetic correlation across LC and CMTs corresponds to Figure 2.

(A) The heatmap showing genetic correlation calculated by LDSC. The color and the number inside the squares indicate the correlation. The statistical significance is denoted by asterisks: * indicates $p < 0.05$; ** indicates FDR-adjusted p (Padj) < 0.05.

(B) The bidirectional causal relationship between LC and CMTs determined through MR analysis.

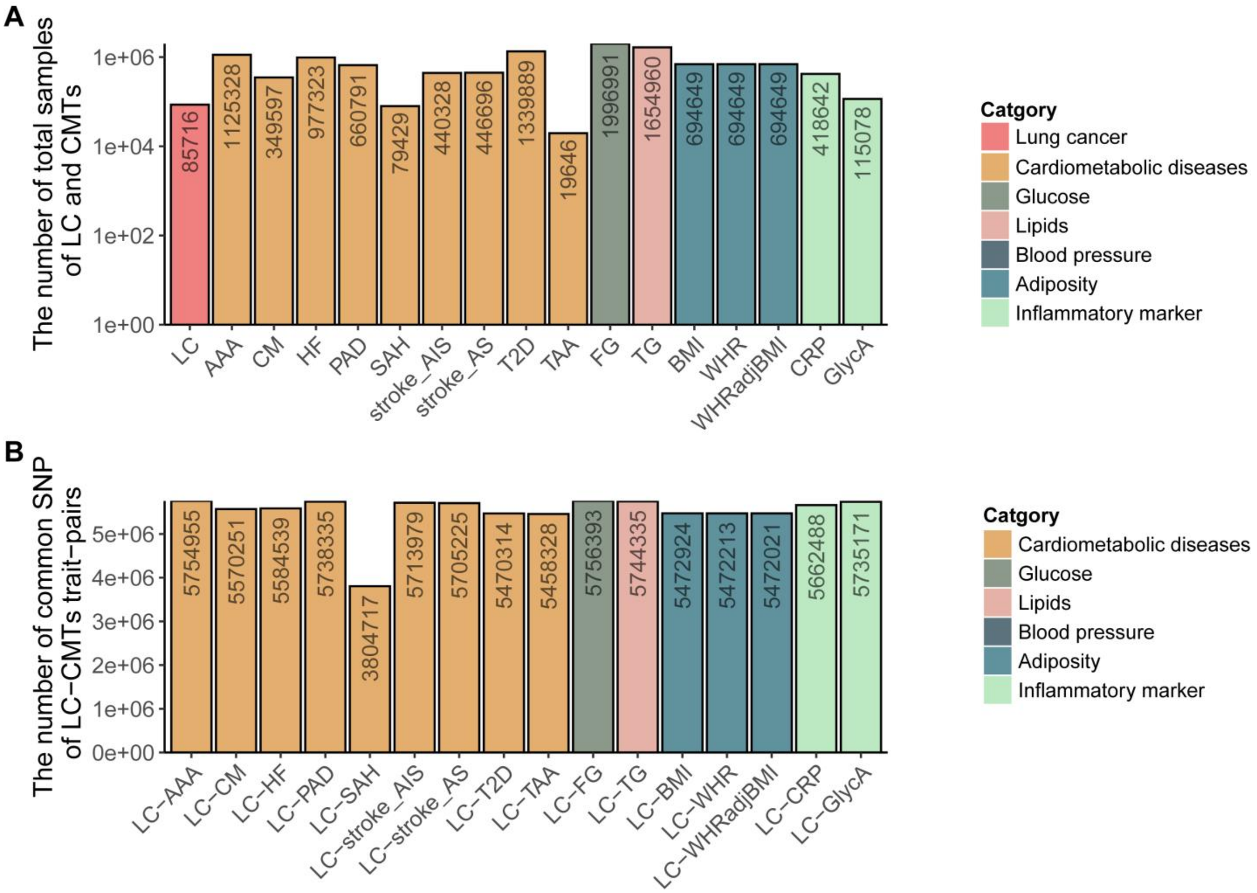

Supplementary Figure 2. Dataset characteristics for genetic analysis of LC and CMTs.

(A) Sample size distribution across GWAS studies for each trait.

(B) Number of valid common SNPs for each LC-CMTs trait pair.

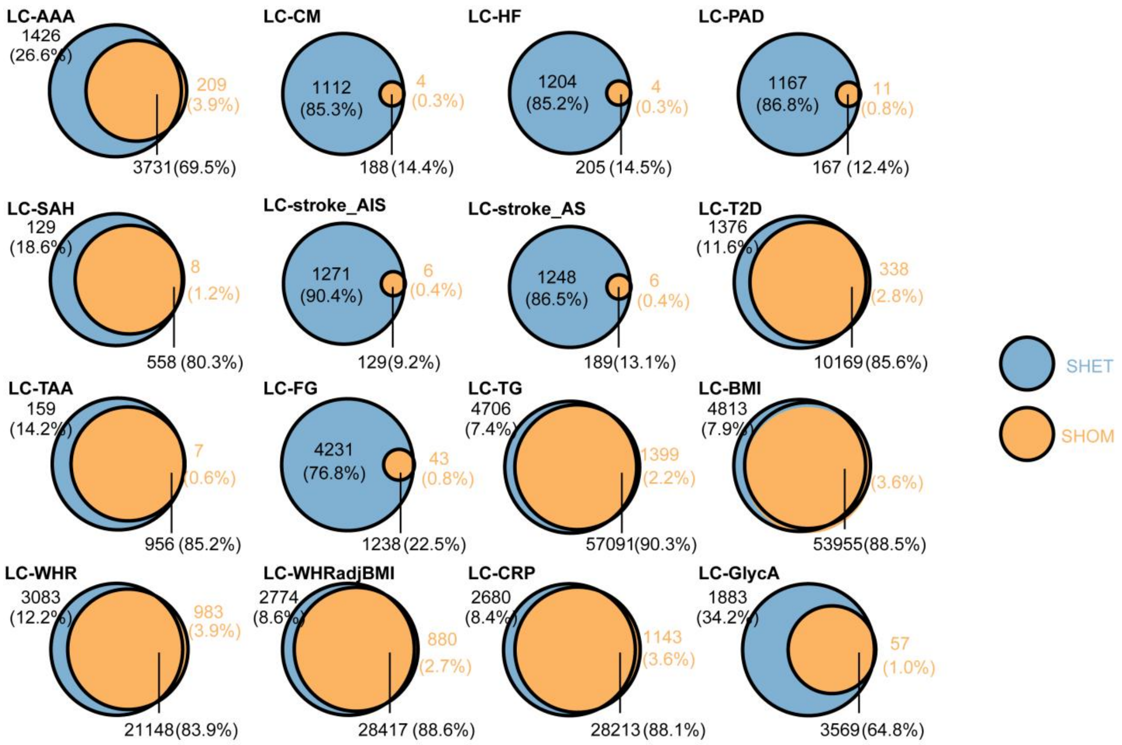

Supplementary Figure 3. Venn diagrams showing the overlap of significant SNPs identified by SHet and SHom methods across 16 LC-CMTs trait pairs.

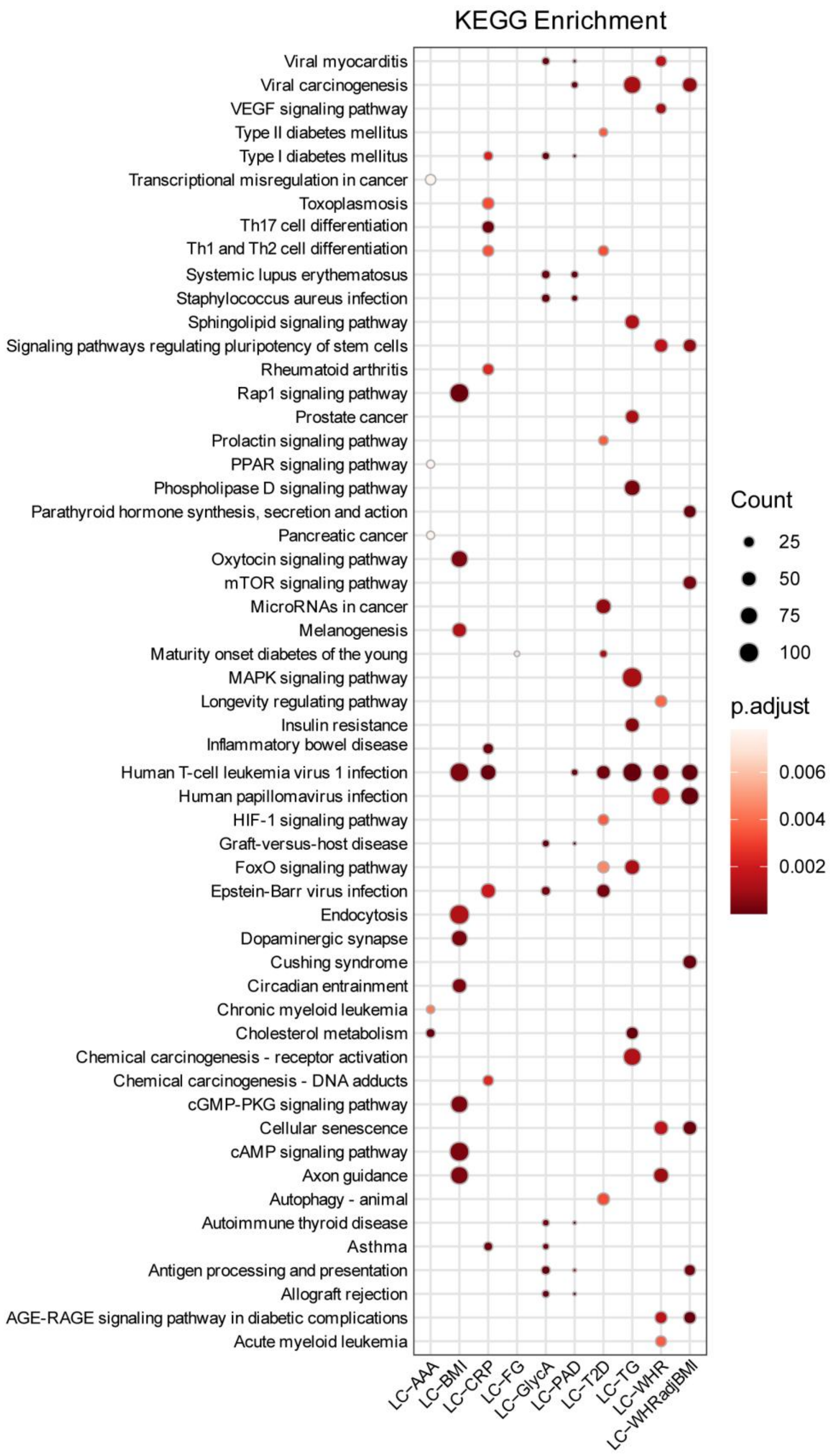

Supplementary Figure 4. KEGG pathway enrichment of the shared genes between LC and CMTs. Only the top 10 enriched pathways passing the hypergeometric test $P < 0.05$ in each trait pair were included.

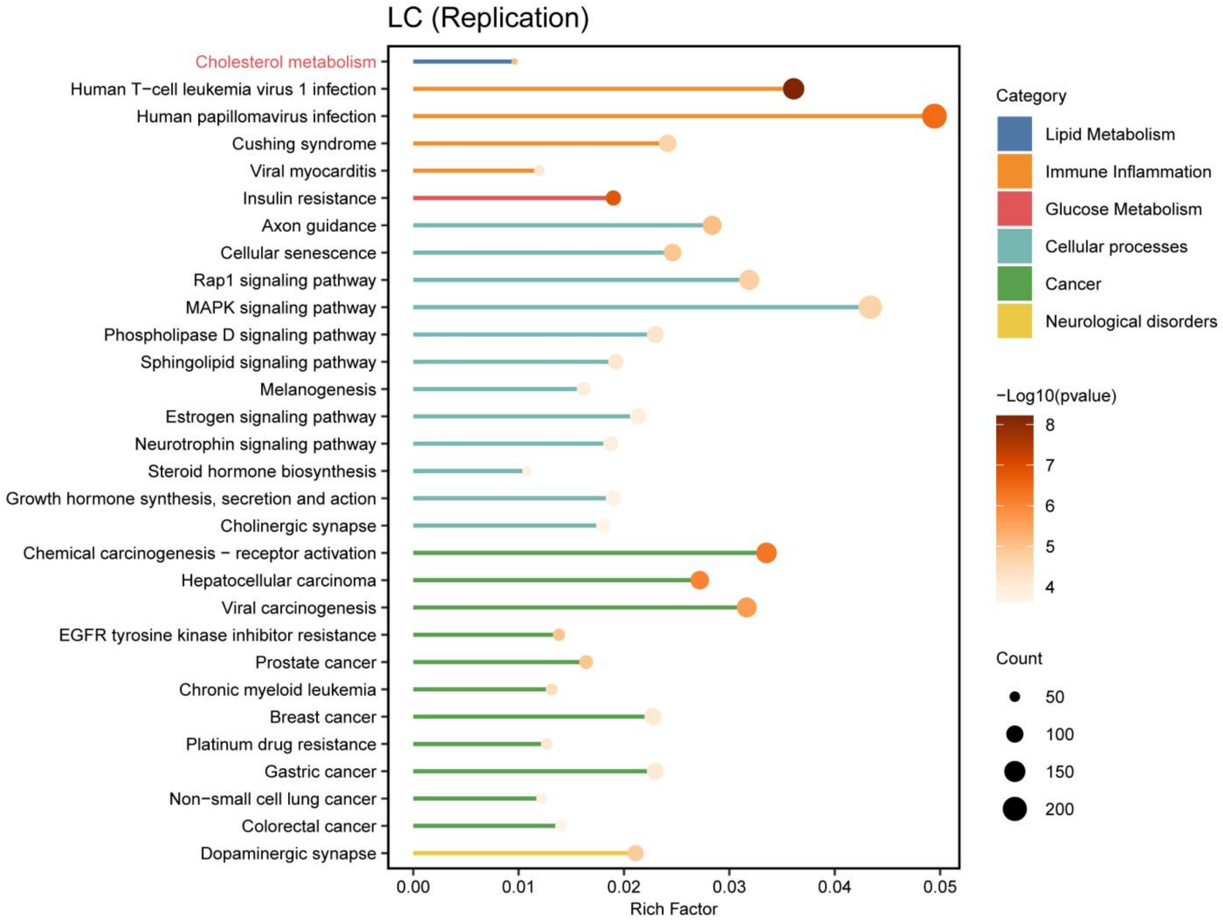

Supplementary Figure 5. KEGG enrichment analysis of the collection of shared genes, arranged according to biological processes. Signaling pathways associated with cholesterol metabolism are highlighted in red.

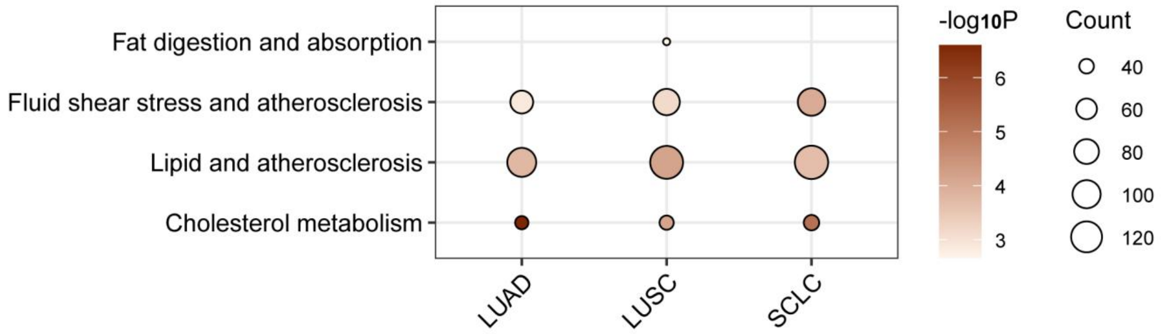

Supplementary Figure 6. Results of lipid and cholesterol metabolism-related pathways in KEGG enrichment of shared genes between LC subtypes and CMTs. LUAD, lung adenocarcinoma; LUSC, lung squamous cell carcinoma; SCLC, small cell lung cancer.

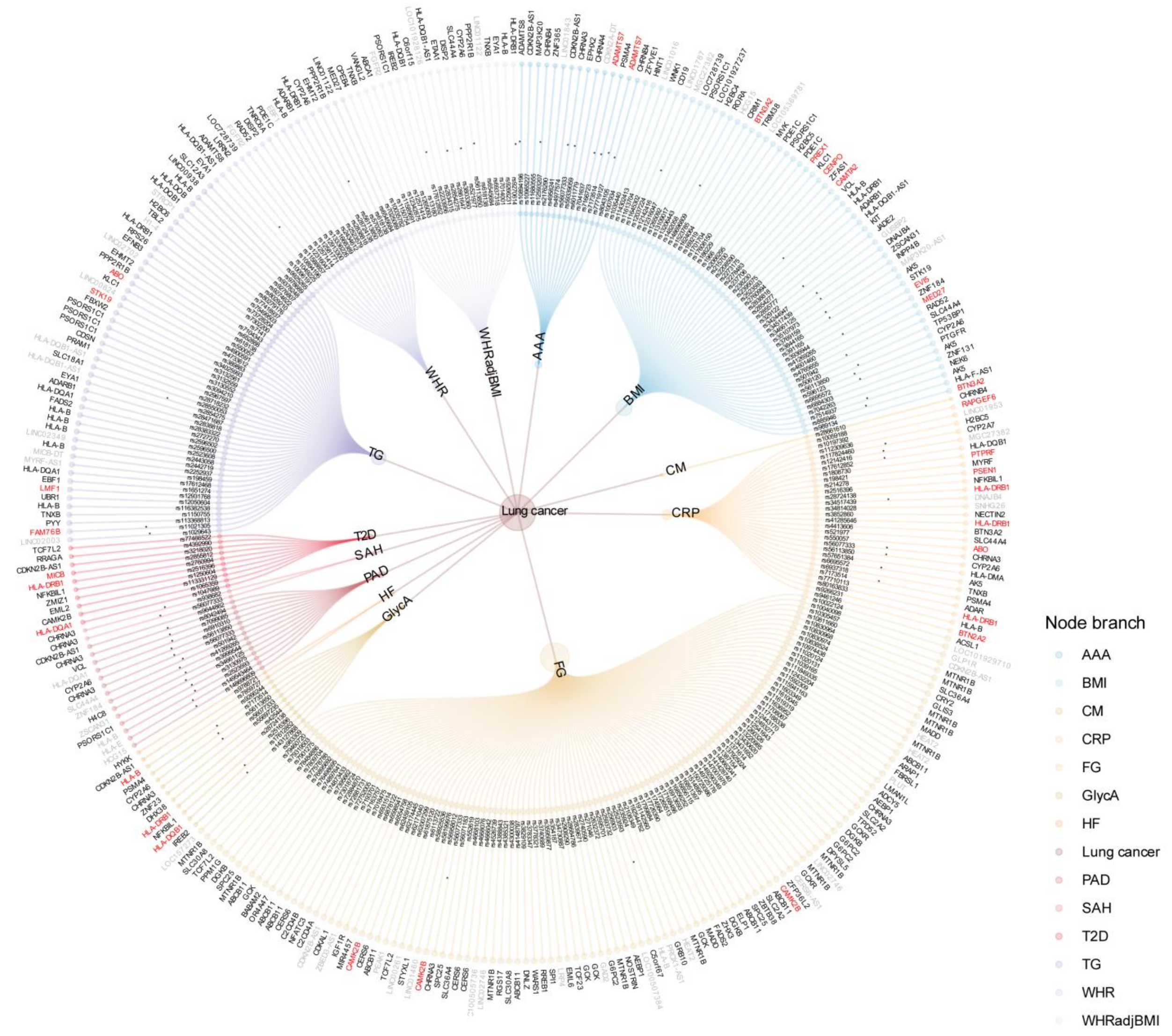

Supplementary Figure 7. Circular dendrograms illustrating the shared loci for LC and CMTs. The inner circle displays independent variants related to each LC-trait pair, with shared causal variants identified by coloc marked with asterisks (PP.H4 > 0.7). The outer circle depicts the genes associated with the shared variants as determined by ANNOVAR. Colors are used to represent the overlap with the four gene identification methodologies: GCTA-fastBAT, MAGMA, TWAS, and SMR. Genes not identified by any method are shown in gray, those identified by at least one method are in black, and those recognized by all four methods are highlighted in red.

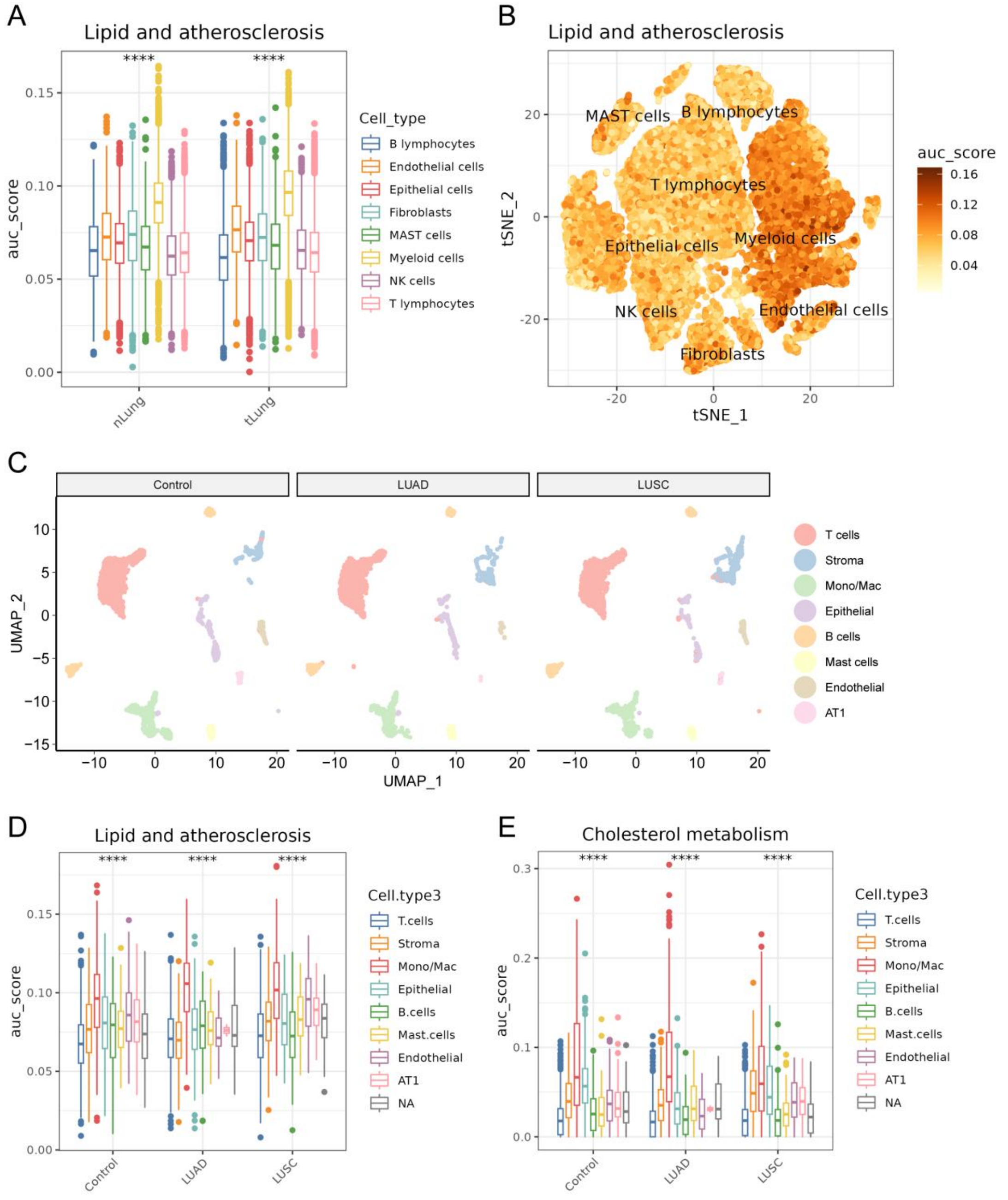

Supplementary Figure 8. Enhanced cholesterol metabolism of mo-mac in LUAD.

(A) The boxplot showing the scores of lipid and atherosclerosis gene sets by tissue origin and cell subsets.

(B) tSNE plot colored by the scores of lipid and atherosclerosis gene sets in tlung. nlung, normal tissues from lungs; tlung, cancer tissue from lungs.

(C) In the validation cohort of lung cancer, the tSNE plot of single cells colored by the major cell lineages, color-coded by clusters and cell subsets as indicated.

(D-E) The boxplot showing the scores of the lipid and atherosclerosis and cholesterol metabolism gene sets by tissue origin and cell subsets.

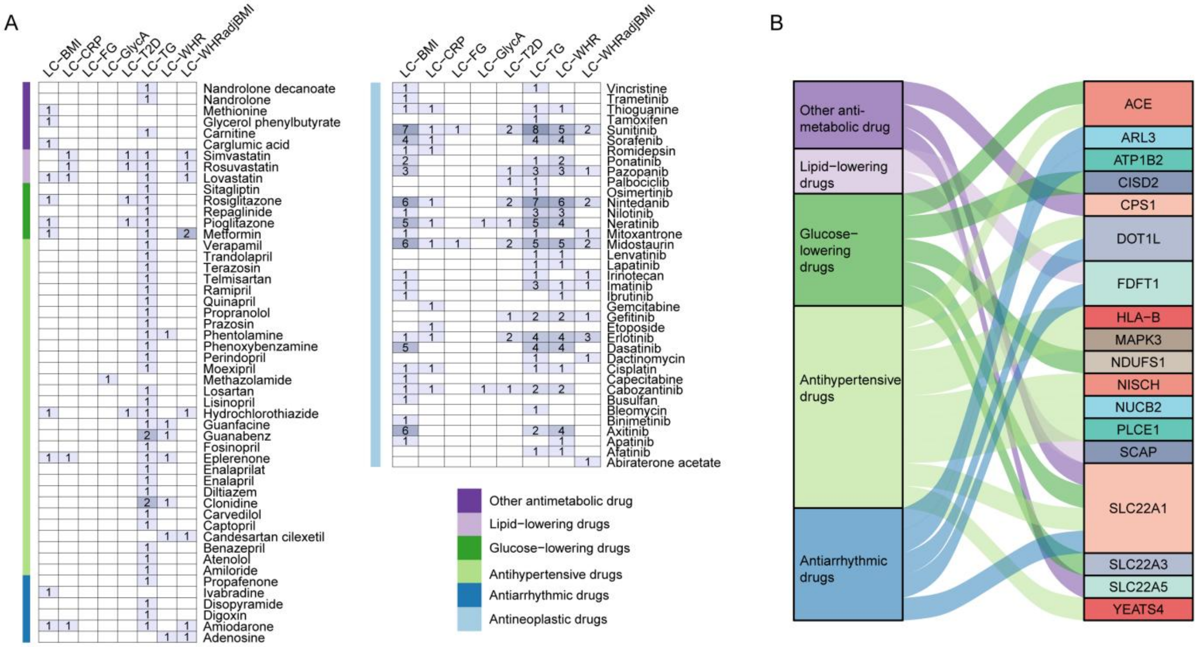

Supplementary Figure 9. Matching between disease pathological pathways, inferred from shared genes for each trait pair, and drug pharmacological pathways.

(A) The number of targets of the drug grouped by LC-trait pair.

(B) Sankey diagram of drug categories and corresponding targets.
